# Wellbeing, quality of life, presence of concurrent diseases, and survival times in untreated and treated German Shepherd dogs with dwarfism

**DOI:** 10.1371/journal.pone.0255678

**Published:** 2021-08-09

**Authors:** Stefanie Kitzmann, Katrin Hartmann, Yury Zablotski, Anna Rieger, Ralf Mueller, Astrid Wehner

**Affiliations:** Center of Clinical Veterinary Medicine, Clinic of Small Animal Medicine, Ludwig Maximilian University, Munich, Germany; University of Pisa, ITALY

## Abstract

**Background:**

Pituitary dwarfism (PD) in German Shepherd dogs (GSD) is a rare endocrinopathy. Cause and inheritance of the disease are well characterized, but the overall survival time, presence of concurrent diseases, quality of life (QoL) and influence of different treatment options on those parameters is still not well investigated. The aim of this study was to obtain data regarding the disease pattern of GSD with PD and to investigate the impact of treatment.

**Methods:**

47 dogs with dwarfism (presumably PD) and 94 unaffected GSD serving as controls were enrolled. Data were collected via a standardized questionnaire, which every owner of a participating dog had completed. Dogs with PD were grouped based on three categories of treatment: Group 1 (untreated), group 2 (treated with levothyroxine), group 3 (treated with thyroxine and progestogens or with growth hormone (GH)). Groups were compared using One-Way-Anova, Kruskal-Wallis test or Wilcoxon-rank-sum test. Categorical analysis was performed using Two-Sample-Chi-Squared-test.

**Results:**

Dogs treated with thyroxine and gestagen or GH were significantly taller and heavier compared to all other dogs with PD. Quality of life was best in dogs with PD treated with thyroxine and similar to unaffected GSD. Treatment increased survival time in dogs with PD independent of the treatment strategy. Dogs receiving thyroxine and progestogens or GH did not develop chronic kidney disease (CKD).

**Conclusion:**

GSD with PD should be treated at least for their secondary hypothyroidism to increase survival time. Additional treatment with progestogens or GH improves body size and seems to protect against the occurrence of CKD.

## Introduction

Failure to grow is defined as not growing at the anticipated rate or to a normal extent. Genetic factors play a major role in linear growth, with numerous genes involved in its regulation. To meet its full genetic potential for growth, any animal must consume sufficient calories and nutrients. Following consumption, food must be digested, absorbed, and the nutrients transported to the necessary tissues and used for metabolic maintenance and growth. The causes of failure to grow can be subdivided into three major groups: inadequate intake of calories and nutrients, metabolic changes associated with increased use of energy, and loss of energy or into endocrine and non-endocrine causes [1].

Pituitary dwarfism (PD) in German Shepherd dogs (GSD) is a simple, autosomal, recessively inherited disease [2, 3], associated with mutations in the LHX3 gene which lead to a defect in the organogenesis of the adenohypophysis [4]. This results in a combined deficiency of growth hormone (GH), thyroid stimulating hormone (TSH) and prolactin (PRL), however the production of adrenocorticotrophic hormone (ACTH) is not impaired [5]. Due to the impaired GH secretion, insulin-like growth factor 1 (IGF-1) in dwarfs is much lower compared to healthy dogs [5, 6]. Diagnosis is established if low GH concentrations after stimulation with GH-releasing hormone (GHRH), ghrelin or an α-adrenergic drug can be demonstrated [7–9]. Low IGF-1 concentrations suggest PD, but overlapping results can occur. Thyroxin (T4) and endogenous TSH are as well decreased [10]. Computed tomography (CT) or magnetic resonance imaging (MRT) can reveal pituitary cysts in many dwarfs [5, 11]. A genetic test is available that identifies dogs that carry the mutation (heterozygote carriers) to exclude them from breeding. However, this test can also be used to identify dogs that are affected by the disease (homozygote animals) [4]. Carriers do not show clinical signs.

The most common reported clinical signs include growth retardation, abnormal hair coat (lack of primary hairs and prominent secondary hairs commonly referred to as `puppy coat`), bilateral symmetrical alopecia and hyperpigmentation of the skin. Many dwarfs develop secondary skin infections. Intrapituitary cysts in the Rathke’s pouch can develop. Glomerular filtration rate can be decreased as GH and IGF-1 are involved in the development of the kidneys. Rats with dwarfism having selective GH deficiency show low glomerular filtration rates in proportion to their low body weights [5], and progressive kidney disease (CKD) has been reported in affected dogs [12–16].

Therapeutic options to treat pituitary dwarfism in GSD include administration of synthetic levothyroxine, porcine GH and medroxyprogesterone acetate (MPA) or proligestone. The latter two can induce expression of the GH gene in the mammary gland [12, 17, 18]. Canine GH is not available for therapeutic use, and treatment with human GH can lead to antibody formation and is therefore not recommended [19].

Prognosis without treatment is assumed to be poor [20], and even with treatment the prognosis is considered guarded [18, 21]. However, larger studies are lacking.

The goal of this study was to investigate the clinical disease pattern in a large number of GSDs with PD, to assess the presence of concurrent disease, wellbeing, quality of life (QoL) and survival time and to compare those results to unaffected GSD. A second study aim was to assess the impact of different treatment options on those parameters.

## Materials and methods

### Ethics and data protection

Ethical approval was obtained from the Ethics Committee of the Faculty of Veterinary Medicine, LMU Munich, Germany (reference number 22-25-04-2018). In the introductory section of the questionnaire it was stated, that dog owners consent to the study once participating. Anonymous participation was possible. If a dog owner provided contact details voluntarily, those data were deleted and data anonymised.

### Data collection

The study was designed as a prospective survey. A standardized web-based questionnaire was developed, which was accessible online in English and German (S1 File). Owners of affected and non-affected GSD´s were invited to participate. An introductory letter containing the link to the study website was available on the homepage of the Clinic of Small Animal Medicine (Ludwig Maximilian University, Munich) and was spread via Facebook and also sent via e-mail to Universities in different countries in Europe. The survey was published online using LimeSurvey (www.limesurvey.org) from April 2018 to July 2018.

The questionnaire consisted of two parts: a general part, which had to be completed by all participants regardless of the dogs`body size, and a second part only for owners of dogs affected by severe dwarfism. The general part assessed signalment, shoulder height, body weight, concurrent diseases, wellbeing (food intake, playing behaviour, fitness), QoL, and in case the dog was already deceased survival times (time of death and cause of death). The second part assessed the performed diagnostic procedures (e.g., genetic testing, measurement of GH, thyroid hormones or IGF-1), the performed treatment (e.g., supplementation of levothyroxine, GH or progestogens), and the impact of treatment on skin and hair condition, body growth, wellbeing, QoL, as well as any adverse effects.

A 6-point rating scale (1–6) was given for most questions regarding wellbeing, fitness and QoL (6 was equivalent to ‘very good’ and 1 to ‘not at all’). To assess changes associated with treatment, a 3- (-1, 0, 1) or 5-point rating scale (-2, -1, 0, 1, 2) was used (-2 = lot worse, -1 = a little worse, 0 = no change, 1 = a little better, 2 = much better). Dichotomous response options (‘yes‘ or ‘no‘) were provided for the assessment of concurrent diseases, performed diagnostics and treatment, as well as for the presence of adverse effects. Survival since diagnosis was grouped as follows: 1 = < 1 year survival; 2 = 1–2 year survival; 3 = 2–3 year survival; 4 = 4–5 year survival; 5 = > 5 year survival. Free text fields were given to allow the owners to expand their answers if needed.

### Animals

47 GSD with dwarfism and 94 unaffected GSD were enrolled. Table 1 gives an overview of the demographic distribution of dogs.

**Table 1:** Demographic distribution of owners filling in the questionnaire.

Country	Controls (n)	Dwarfs (n)
Germany	53	9
Netherlands		8
USA	3	8
United Kingdom		6
Australia		5
Canada		3
Brazil		1
New Zealand		1
South Africa		1
No specification	38	5

Owners of GSH dogs with a normal body size could participate (Controls) as well as dogs with dwarfism (Dwarfs).

Diagnosis of PD was based on the typical clinical presentation (small body size). It had to be expressed by a veterinarian. In most dwarfs, additional tests such as genetic testing, measurement of GH, thyroxine, IGF-1, and GH stimulation testing were performed. Table 2 gives an overview of diagnostic findings in dwarfs. Of the nine dogs in group 2, where only T4 was measured, four dogs had additional clinical findings to suggest PD: 1 dog never had an adult hair coat, one had worsening alopecia, one had alopecia and a heart murmur and one had a Patent Ductus Arteriosus (PDA).

**Table 2:** Diagnostic findings in dwarfs to support a diagnosis of Pituitary Dwarfism (PD) in untreated dwarfs (group 1), dwarfs treated with levothyroxine (group 2), and dwarfs treated with levothyroxine and progestogens or GH (group 3).

Diagnostic findings	Group 1	Group 2	Group 3
	n = 12	n = 22	n = 13
T4^1^	1	9	
Genetics^2^		1	4
Genetics + T4		1	3
GH^3^		2	1
GH + T4		2	1
Genetics + IGF-1^4^ + T4		2	
Genetics + GH + T4			1
Genetics + GH			1
GH-ST^5^+ IGF-1+ T4			1
Gentics + GH-ST + IGF-1 + T4			1
PD^6^ post mortem confirmed	4		
Incomplete ossification of atlas		1	
No testing or other findings suggestive of PD	7	4	

^1^T4 = thyroxine concentration

^2^Genetics = genetic test assessing the mutation in the LHX3 gene

^3^GH = growth hormone

^4^IGF-1 = insulin-like growth factor 1

^5^GH-ST = growth hormone stimulation test

^6^PD = pituitary dwarfism.

GSD with PD were split into three different groups based on the treatment: untreated dwarfs (n = 12, group 1), treated with levothyroxine (n = 22, group 2) and dwarfs treated with levothyroxine and progestogens or GH (n = 13, group 3*)*. In this study, 7 dogs were reported to be treated with porcine GH and levothyroxine. Progestogens are capable to induce expression of the GH gene in the canine mammary gland and ensures subsequent secretion of this GH into the systemic circulation [22]. In this study, 6 dogs were treated with progestogens and levothyroxine. The medication was specified in two dogs (one was treated with medroyprogesterone acetat and one with proligestone). Although, there are differences in the treatment strategies as in the latter GH concentrations never exceed the reference interval [12] and more side effects due to the action of progestons are reported, both groups of dogs were taken into one group as an increase of GH was ensured and to allocate more than 10 individuals to one group.

GSD with a normal stature served as a control group (n = 94; group 0). Dogs with insufficient data were not included.

### Statistical analysis

R 3.6.3 statistical package (R Core Team, 2020) was used for statistical analysis. Normality of data distribution was tested by Shapiro-Wilk test. Two groups were compared using Student´s t-test for normally distributed data and similar variance and Welch`s t-test for normally distributed data and differing variances. Wilcoxon-rank-sum test was used for not normally distributed data. Three or more groups were compared using Fisher`s Anova if data were normally distributed and had similar variances and Welch`s Anova if differing variances were present. Kruskal-Wallis test was used if data were not normally distributed. Post-hoc tests were Dunn test for the parameters age, wellbeing and QoL, Games -Howell test or Student`s t-test for the parameters body weight and heights. Categorical analysis was performed using two-Sample-Chi-Squared test. Pairwise Fisher’s exact test was used as post-hoc test. Inside every group one-Sample-Chi-Squared-Goodness-Of-Fit test was used to check whether counts of 1 and 2 (‘yes’ or ‘no’) differ. Survival analysis in dwarfs was performed using Kaplan-Meier. Comparison between groups was performed by using Log-Rank test.

## Results

### Signalment, concurrent diseases and survival times

**Fig 1:**
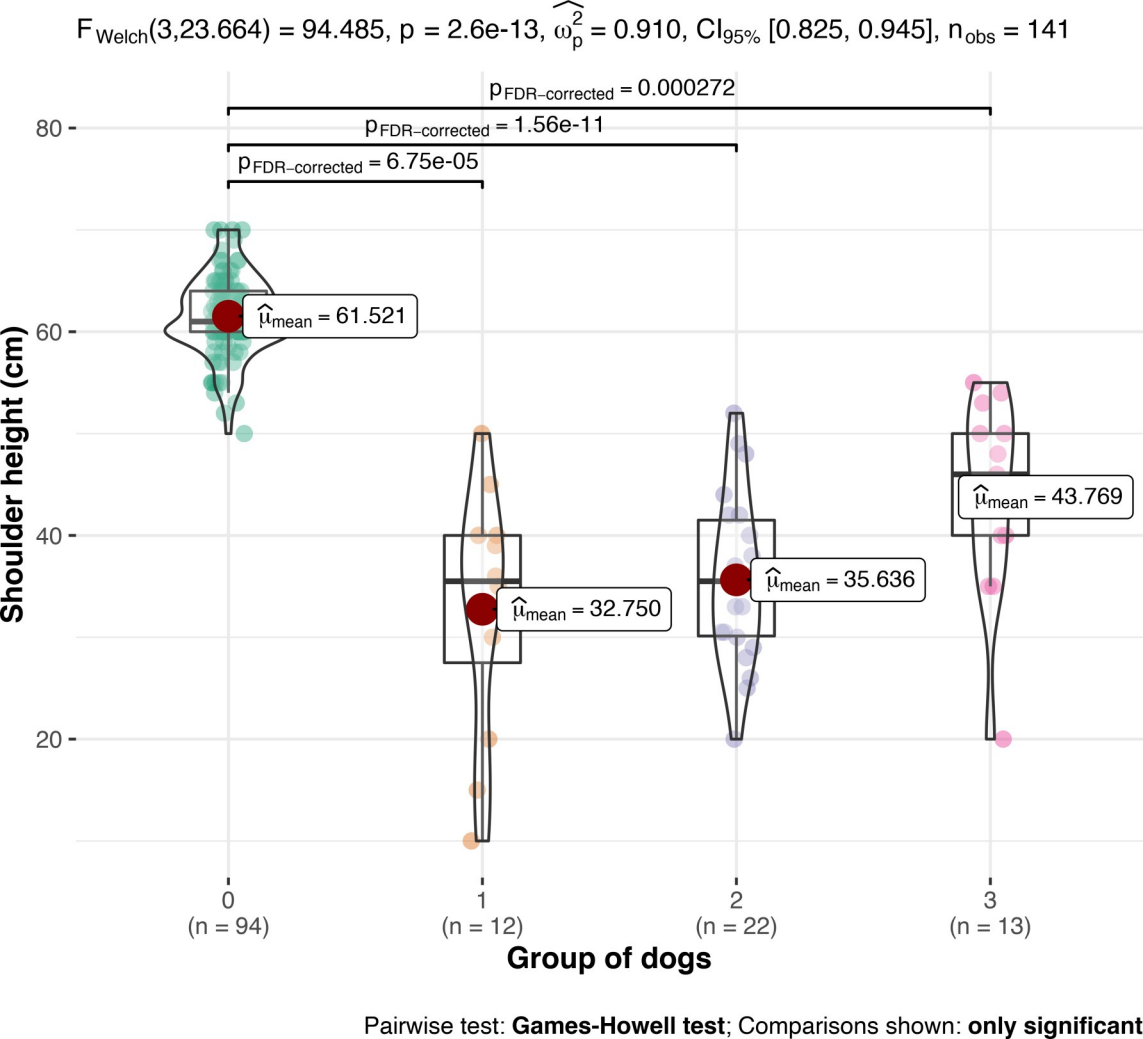
Shoulder height (cm) in control GSD (group 0), untreated dwarfs (group 1), dwarfs treated with levothyroxine (group 2), and dwarfs treated with levothyroxine and gestagens or GH (group 3). Comparison between groups was performed by using one-Way-Anova.

Results of signalment, shoulder height, body weight, concurrent diseases, and survival times of all groups are shown in Table 3 and Fig 1.

**Table 3:** Description of the population, presence of concurrent diseases and survival times in control GSD (group 0), untreated dwarfs (group 1), dwarfs treated with levothyroxine (group 2), and dwarfs treated with levothyroxine and progestogens or GH (group 3). Dichotomous response options (‘yes‘ or ‘no‘) were provided for the assessment of sex, adulthood, stature and concurrent diseases. Survival since diagnosis was grouped as follows: 1 = < 1 year survival; 2 = 1–2 year survival; 3 = 2–3 year survival; 4 = 4–5 year survival; 5 = > 5 year survival. Comparison between groups was performed by using one-way-ANOVA or Kruskal-Wallis test for numerical data and Chi-square two sample test for categorical data.

	Group 0	Group 1	Group 2	Group 3	Comparison between groups p-value^2^
	n^1^ = 94	n = 12	n = 22	n = 13	
	p-value (if `yes`or `no`)	p-value (if `yes`or `no`)	p-value (if `yes`or `no`)	p-value (if `yes`or `no`)	
**Sex (male)**	yes (male): 37/94 (39%)	yes (male): 7/12 (58%)	yes (male): 9/22 (41%)	yes (male): 6/13 (46%)	*chi-square two sample test*:
	no (female): 57/94 (61%)	no (female): 5/12 (42%)	no (female): 13/22 (59%)	no (female): 7/13 (54%)	
	*chi-square one sample goodness-of-fit test*: ***0*.*039***^3^	*chi-square one sample goodness-of-fit test*: *0*.*564*	*chi-square one sample goodness-of-fit test*: *0*.*394*	*chi-square one sample goodness-of-fit test*: *0*.*782*	*0*.*640*
**Age at time of entering the study (days)**	mean: 2176.9	mean: 854.9	mean: 2059.1	mean: 2027.3	*Kruskal-Wallis test*: ***0*.*008***
	dead: 12 dogs	dead: 5 dogs	dead: 4 dogs	dead: 5 dogs	
	alive: 84 dogs	alive: 7 dogs	alive: 18 dogs	alive: 8 dogs	*Dunn test*:
					***0*** *vs* ^4^ *1 =* ***0*.*004***
					*1 vs* ***2*** *=* ***0*.*015***
					*1 vs* ***3*** *=* ***0*.*015***
**Adult (dogs > 18 months)**	yes: 87/94 (93%)	yes: 5/12 (42%)	yes: 20/22 (91%)	yes: 11/13 (85%)	*chi-square two sample test*:
	no: 7/94 (7%)	no: 7/12 (58%)	no: 2/22 (9%)	no: 2/13 (15%)	
	*chi-square one sample goodness-of-fit test*: ***≤0*.*001***	*chi-square one sample goodness-of-fit test*: *0*.*564*	*chi-square one sample goodness-of-fit test*: ***≤0*.*001***	*chi-square one sample goodness-of-fit test*: ***0*.*013***	***<0*.*001***
					*pairwise Fisher`s exact test*:
					***0*** *vs 1 =* ***<0*.*001***
					***1*** *vs 2 =* ***0*.*004***
					***1*** *vs 3 =* ***0*.*041***
**Proportionate small stature**	-	yes: 4/12 (33%)	yes: 17/22 (77%)	yes: 12/13 (92%)	*chi-square two sample test*:
		no: 8/12 (67%)	no: 5/22 (23%)	no: 1/13 (8%)	
					***0*.*003***
		*chi-square one sample goodness-of-fit test*: *0*.*248*	*chi-square one sample goodness-of-fit test*: ***0*.*011***	*chi-square one sample goodness-of-fit test*: ***0*.*002***	*pairwise Fisher`s exact test*:
					*1 vs* ***2*** *=* ***0*.*025***
					*1 vs* ***3*** *=* ***0*.*004***
**Body weight (kg)**	mean: 33.1	mean: 10.5	mean: 12.7	mean: 17.8	*Fisher´s ANOVA test*: ***<0*.*001***
					*Student`s t-test*:
					***0****vs 1*, *2*, *3 =****≤0*.*001***
					*1 vs* ***3*** *=* ***0*.*001***
					*2 vs* ***3*** *=* ***0*.*005***
					*if analysis is performed without group 0*:
					*Fisher`s ANOVA test*: ***0*.*019***
					*Student`s t-test*:
					*1 vs* ***3*** *=* ***0*.*011***
					*2 vs* ***3*** *=* ***0*.*029***
**Shoulder height (cm)**	mean: 61.5	mean: 32.8	mean: 35.6	mean: 43.8	*Welch-ANOVA test*: ***<0*.*001***
					*Games-Howell test*:
					***0****vs 1*, *2*, *3 =****≤0*.*001***
					*if analysis is performed without group 0*:
					*Fisher´s ANOVA test*: ***0*.*017***
					*Student´s t-test*:
					*1 vs* ***3*** *=* ***0*.*022***
					*2 vs* ***3*** *=* ***0*.*033***
**Orthopedic disease**	yes: 21/94 (22%)	yes: 2/12 (17%)	yes: 3/22 (14%)	yes: 6/13 (46%)	*chi-square two sample test*: *0*.*147*
	no: 73/94 (78%)	no: 10/12 (83%)	no: 19/22 (86%)	no: 7/13 (54%)	
	*chi-square one sample goodness-of-fit test*: ***<0*.*001***	*chi-square one sample goodness-of-fit test*: ***<0*.*05***	*chi-square one sample goodness-of-fit test*: ***<0*.*001***	*chi-square one sample goodness-of-fit test*: *n*.*s*.^5^	
**Dermatological disease**	yes: 14/94 (15%)	yes: 4/12 (33%)	yes: 14/22 (64%)	yes: 5/13 (38%)	*chi-square two sample test*: ***<0*.*001***
	no: 80/94 (85%)	no: 8/12 (67%)	no: 8/22 (36%)	no: 8/13 (62%)	
	*chi-square one sample goodness-of-fit test*: ***<0*.*001***	*chi-square one sample goodness-of-fit test*: *n*.*s*.	*chi-square one sample goodness-of-fit test*: *n*.*s*.	*chi-square one sample goodness-of-fit test*: *n*.*s*.	*pairwise Fisher`s exact test*:*0 vs****2****=****<0*.*001***
**Ophtalmologic disease**	yes: 1/94 (1%)	yes: 0/12 (0%)	yes: 2/22 (9%)	yes: 1/13 (8%)	*chi-square two sample test*: *0*.*130*
	no: 93/94 (99%)	no: 12/12 (100%)	no: 20/22 (91%)	no: 12/13 (92%)	
	*chi-square one sample goodness-of-fit test*: ***<0*.*001***	*chi-square one sample goodness-of-fit test*: ***<0*.*001***	*chi-square one sample goodness-of-fit test*: ***<0*.*001***	*chi-square one sample goodness-of-fit test*:***<0*.*01***	
**Pancreatic insufficiency**	yes: 1/94 (1%)	yes: 0/12 (0%)	yes: 0/22 (0%)	yes: 0/13 (0%)	*chi-square two sample test*: *0*.*918*
	no: 93/94 (99%)	no: 12/12 (100%)	no: 22/22 (100%)	no: 13/13 (100%)	
	*chi-square one sample goodness-of-fit test*: ***<0*.*001***	*chi- one sample goodness-of-fit test*: ***<0*.*001***	*chi-square one sample goodness-of-fit test*: ***<0*.*001***	*chi-square one sample goodness-of-fit test*:***<0*.*001***	
**Chronic gastrointestinal disease**	yes: 4/94 (4%)	yes: 1/12 (8%)	yes: 3/22 (14%)	yes: 0/13 (0%)	*chi-square two sample test*: *0*.*272*
	no: 90/94 (96%)	no: 11/12 (92%)	no: 19/22 (86%)	no: 13/13 (100%)	
	*chi-square one sample goodness-of-fit test*:***<0*.*001***	*chi-square one sample goodness-of-fit test*:***<0*.*01***	*chi-square one sample goodness-of-fit test*:***<0*.*001***	*chi-square one sample goodness-of-fit test*: ***<0*.*001***	
**Neurological disease**	yes: 0/94 (0%)	yes: 0/12 (0%)	yes: 2/22 (9%)	yes: 1/13 (8%)	*chi-square two sample test*: ***0*.*025***
	no: 94/94 (100%)	no: 12/12 (100%)	no: 20/22 (91%)	no: 12/13 (92%)	*pairwise Fisher`s exact test*:
	*chi-square one sample goodness-of-fit test*: ***<0*.*001***	*chi-square one sample goodness-of-fit test*: ***<0*.*001***	*chi-square one sample goodness-of-fit test*: ***<0*.*001***	*chi-square one sample goodness-of-fit test*: ***<0*.*01***	*0 vs* ***2*** *=* ***0*.*035***
**Chronic kidney disease**	yes: 0/94 (0%)	yes: 2/12 (17%)	yes: 2/22 (9%)	yes: 0/13 (0%)	*chi-square two sample test*: ***0*.*002***
	no: 94/94 (100%)	no: 10/12 (83%)	no: 20/22 (91%)	no: 13/13 (100%)	*pairwise Fisher`s exact test*:
					*0 vs* ***1*** *=* ***0*.*012***
					*0 vs* ***2*** *=* ***0*.*035***
	*chi-square one sample goodness-of-fit test*: ***<0*.*001***	*chi-square one sample goodness-of-fit test*: ***<0*.*05***	*chi-square one sample goodness-of-fit test*: ***<0*.*001***	*chi-square one sample goodness-of-fit test*: ***<0*.*001***	
**Lower urinary or genital disease**	yes: 4/94 (4%)	yes: 0/12 (0%)	yes: 0/22 (0%)	yes: 1/13 (8%)	*chi-square two sample test*: *0*.*564*
	no: 90/94 (96%)	no: 12/12 (100%)	no: 22/22 (100%)	no: 12/13 (92%)	
	*chi-square one sample goodness-of-fit test*: ***<0*.*001***	*chi-square one sample goodness-of-fit test*: ***<0*.*001***	*chi-square one sample goodness-of-fit test*: ***<0*.*001***	*chi-square one sample goodness-of-fit test*: ***<0*.*01***	
**Neoplastic disease**	yes: 10/94 (11%)	yes: 0/12 (0%)	yes: 1/22 (5%)	yes: 0/13 (0%)	*chi-square two sample test*: *0*.*322*
	no: 84/94 (89%)	no: 12/12 (100%)	no: 21/22 (95%)	no: 13/13 (100%)	
	*chi-square one sample goodness-of-fit test*: ***<0*.*001***	*chi-square one sample goodness-of-fit test*: ***<0*.*001***	*chi-square one sample goodness-of-fit test*: ***<0*.*001***	*chi-square one sample goodness-of-fit test*: ***<0*.*001***	
**Cardiac disease**	yes: 5/94 (5%)	yes: 0/12 (0%)	yes: 3/22 (14%)	yes: 3/13 (23%)	*chi-square two sample test*: *0*.*069*
	no: 89/94 (95%)	no: 12/12 (100%)	no: 19/22 (86%)	no: 10/13 (77%)	
	*chi-square one sample goodness-of-fit test*: ***<0*.*001***	*chi-square one sample goodness-of-fit test*: ***<0*.*001***	*chi-square one sample goodness-of-fit test*: ***<0*.*001***	*chi-square one sample goodness-of-fit test*: *n*.*s*.	
**Other endocrine disease (besides PD)**	yes: 4/94 (4%)	yes: 0/12 (0%)	yes: 0/22 (0%)	yes: 0/13 (0%)	*chi-square two sample test*: ***<0*.*001 (group 0)***
	no: 90/94 (96%)	no: 12/12 (100%)	no: 22/22 (100%)	no: 13/13 (100%)	
	*chi-square one sample goodness-of-fit test*: ***<0*.*001***	*chi-square one sample goodness-of-fit test*: ***<0*.*001***	*chi-square one sample goodness-of-fit test*: ***<0*.*001***	*chi-square one sample goodness-of-fit test*: ***<0*.*001***	
**Respiratory disease**	yes: 2/94 (2%)	yes: 0/12 (0%)	yes: 1/22 (5%)	yes: 0/13 (0%)	*chi-square two sample test*: *0*.*762*
	no: 92/94 (98%)	no: 12/12 (100%)	no: 21/22 (95%)	no: 13/13 (100%)	
	*chi-square one sample goodness-of-fit test*: ***<0*.*001***	*chi-square one sample goodness-of-fit test*: ***<0*.*001***	*chi-square one sample goodness-of-fit test*: ***<0*.*001***	*chi-square one sample goodness-of-fit test*: ***<0*.*001***	
**Survival since diagnosis of PD (1–6)**		mean: 2.33	mean: 4.27	mean: 4.85	*Kruskal-Wallis test*: ***0*.*005***
					*Dunn test*:
					*1 vs* ***2*** *=* ***0*.*011***
					*1 vs* ***3*** *=* ***0*.*007***

^1^n = number

^2^p = level of significance

^3^numbers or groups marked in bold indicate statistical significance

^4^vs = versus

^5^n.s. = not significant.

Regarding dermatologic disease, there were significant differences between groups and especially dwarfs of group 2 had more dermatological problems such as pyoderma (3/22 patients, 13.6%) and alopecia (3/22 patients, 13.6%) than controls, where (2/94 patients, 2.1%) had pyoderma (S1 Table).

Regarding orthopedic diseases, 4 of 13 (30.8%) dwarfs in group 3 suffered from incomplete ossification of the neck. Six of 94 (6.4%) controls (group 0) and only one dwarf (group 3) had degenerative joint disease (DJD). In 9/94 (9.6%) dogs of the control population the orthopedic problem was not specified. Regarding cardiac diseases, 3/13 (23.1%) patients in group 3 and 1/22 (4.5%) of group 2 suffered from a PDA. One of 22 dwarf in group 2 had a heart murmur, but its etiology was unknown. 10/94 (10.6%) control dogs and only one dwarf (group 2) were diagnosed with various malignant neoplasias. Respiratory, urogenital, neurologic, endocrine (besides dwarfism), GI disease, EPI and ophtalmologic problems did not occur frequently in all groups of dogs (S1 Table).

Results of wellbeing (food intake, playful behaviour, fitness), and Quality of life (QoL) in all dogs are given in Table 4.

**Table 4:** Description of parameters assessing wellbeing and quality of life in control GSD (group 0), untreated dwarfs (group 1), dwarfs treated with levothyroxine (group 2), and dwarfs treated with levothyroxine and progestogens or GH (group 3). A 6-point rating scale (1–6) was given for food intake, playing behaviour and quality of life. A higher value was related to a better outcome (6 was equivalent to ‘very good’ and 1 to ‘not at all’). Comparison between groups was performed by using one-way-ANOVA or Kruskal-Wallis test.

	Group 0	Group 1	Group 2	Group 3	Comparison between groups p-value^1^
**Food intake (1–6)**	mean: 5.35	mean: 4.33	mean: 4.41	mean: 5.08	*Kruskal-Wallis test*: ***0*.*001***^3^
	n^2^ = 94	n = 12	n = 22	n = 13	*Dunn test*:*0 vs*^4^*2 =****0*.*001***
**Playful behaviour (1–6)**	mean: 5.4	mean: 5.25	mean: 5.23	mean: 3.92	*Kruskal-Wallis test*: ***0*.*001***
	n = 94	n = 12	n = 22	n = 13	*Dunn test*:
					*0 vs 3 =* ***≤0*.*001***
					*1 vs 3 =* ***0*.*006***
					*2 vs 3 =* ***0*.*003***
**Quality of life (1–6)**	mean: 5.6	mean: 4.42	mean: 5.41	mean: 4.23	*Kruskal-Wallis test*: ***<0*.*001***
	n = 94	n = 12	n = 22	n = 13	*Dunn test*:
					*0 vs 1 =* ***0*.*001***
					*0 vs 3 =* ***<0*.*001***
					*1 vs 2 =* ***0*.*01***
					*2 vs 3 =* ***0*.*001***
**Mean walking duration/24 hrs (min)**	130.8	70.7	68.6	82.5	*Kruskal-Wallis test*: ***0*.*001***
	n = 90	n = 7	n = 14	n = 12	*Dunn test*:
					*0 vs 1 =* ***0*.*05***
					*0 vs 2 =* ***0*.*008***

^1^p = level of significance

^2^n = number

^3^p-values numbers marked in bold indicate statistical significance

^4^vs = versus.

**Fig 2:**
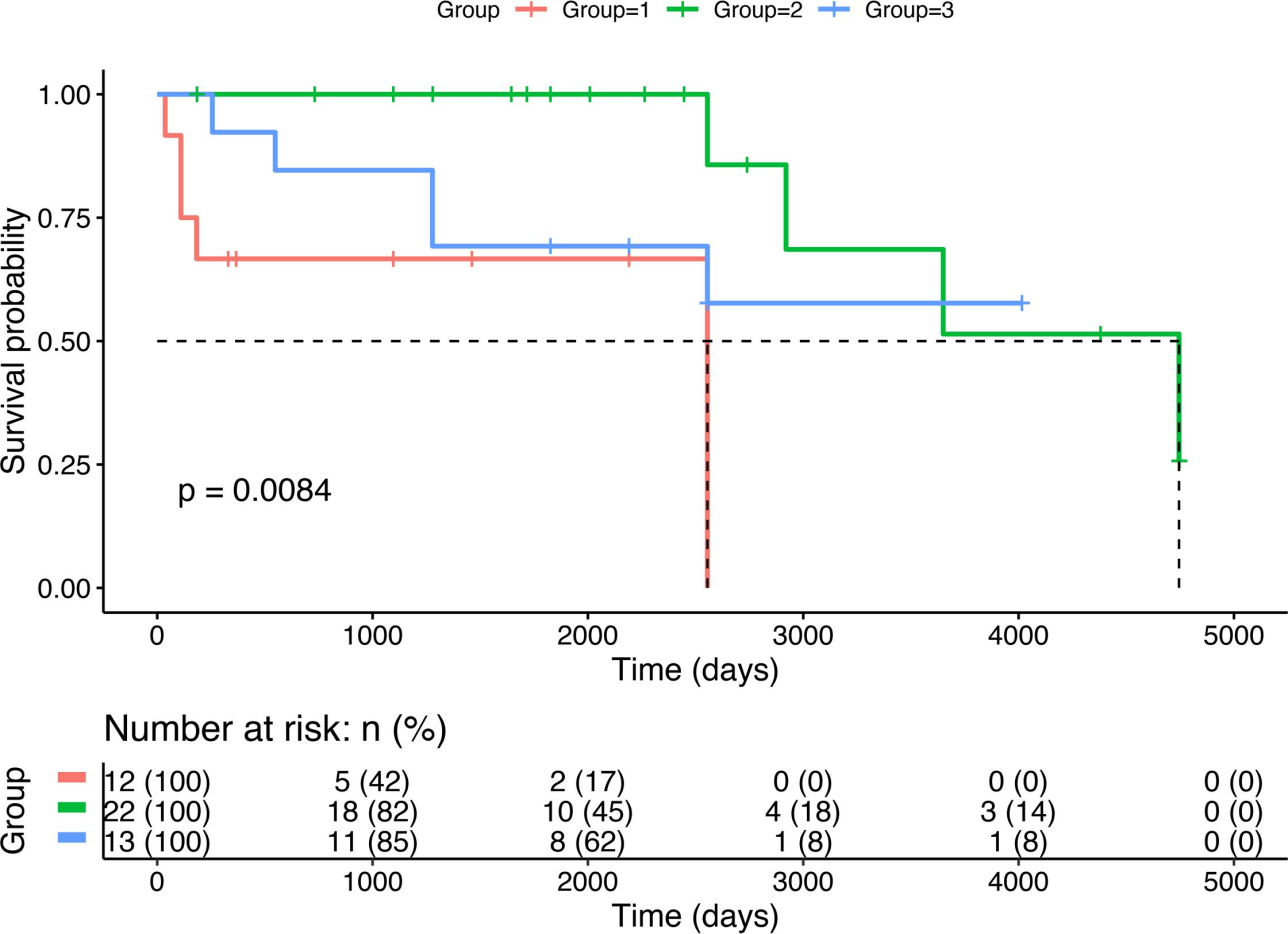
Survival analysis in untreated dwarfs (group 1), dwarfs treated with levothyroxine (group 2), and dwarfs treated with levothyroxine and gestagens or GH (group 3). Comparison between groups was performed by using Kaplan-Meier.

Survival times are shown in Fig 2. Treated dwarfs survived significantly longer compared to untreated dogs. At the time of data collection, 14/47 (29.8%) GSD with PD had died or had been euthanized. 3 of 14 (21.4%) patients that were no longer alive suffered from incomplete ossification of the atlas. Two of 14 (14.3%) dwarfs died due to progressive chronic kidney disease (CKD), including 1/22 patient in group 2 and 1/12 patient in group 1. For four dogs in group 1 the owners reported death or euthanasia due to the severity of the disease itself. In the control group (group 0) 12/94 (12.8%) dogs had died or had been euthanized at the time of data collection. Six of those 12 dogs died or were euthanized because of neoplasia (S2 Table).

### Impact of treatment on skin and hair condition and body growth, wellbeing, QoL, side effects and survival

The effect of treatment on skin and hair condition, body growth, occurrence of adverse effects and time until improvement was assessed in dwarfs of groups 2 and group 3. Results are given in Table 5. In regard to adverse effects, in group 3, those were only seen in 2/6 dogs treated with progestogens (1 dog developed pyometra and mastitis and 1 dog developed mammary tumor and pyoderma). In group 2, 3 dogs were reported to have adverse effects (1 with vaginal discharge due to permanent oestrus and 2 dogs had pyoderma).

**Table 5:** Changes after treatment initiation with levothyroxine (group 2) or levothyroxine and progestogens or GH (group 3). Dichotomous response options (‘yes‘ or ‘no‘) were provided to assess the improvement of hair and skin condition, growth, fitness and side effects. Comparison between groups was performed by using Wilcoxon-rank-sum test or t-test for numerical data and Chi-square two sample test for categorical data.

	Group 2	Group 3	Comparison between groups
	n^1^ = 22	n = 13	
	p-value (if `yes`or `no`)	p-value (if `yes`or `no`)	p-value^2^
**Growth of guard hair**	yes: 18/22 (82%)	yes: 12/13 (92%)	*chi-square two sample test*: *0*.*392*
	no: 4/22 (18%)	no: 1/13 (8%)	
	*chi-square one sample goodness-of-fit test*: ***<0*.*01***^3^	*chi-square one sample goodness-of-fit test*:***<0*.*01***	
**Improvement of skin condition**	yes: 9/22 (41%)	yes: 6/13 (46%)	*chi-square two sample test*: *0*.*762*
	no: 13/22 (59%)	no: 7/13 (54%)	
	*chi-square one sample goodness-of-fit test*: *n*.*s*.^4^	*chi-square one sample goodness-of-fit test*: *n*.*s*.	
**Increasing body size**	yes: 1/22 (5%)	yes: 6/13 (46%)	*chi-square two sample test*: ***0*.*003***
	no: 21/22 (95%)	no: 7/13 (54%)	
	*chi-square one sample goodness-of-fit test*: ***<0*.*001***	*chi-square one sample goodness-of-fit test*: *n*.*s*.	
**Increasing fitness**	yes: 8/22 (36%)	yes: 8/13 (62%)	*chi-square two sample test*: *0*.*149*
	no: 14/22 (64%)	no: 5/13 (38%)	
	*chi-square one sample goodness-of-fit test*: *n*.*s*.	*chi-square one sample goodness-of-fit test*: *n*.*s*.	
**Time period for improvement (days)**	mean: 42	mean: 33.4	*Wilcoxon rank sum test*: *0*.*208*
**Adverse effects**	yes: 3/22 (14%)	yes: 2/13 (15%)	*chi-square two sample test*: *0*.*886*
	no: 19/22 (86%)	no: 11/13 (85%)	
	*chi-square one sample goodness-of-fit test*: ***<0*.*001***	*chi-square one sample goodness-of-fit test*: ***<0*.*05***	

^1^n = number

^2^p = level of significance

^3^p-values numbers marked in bold indicate statistical significance

^4^n.s. = not significant.

Degree of change of the wellbeing and QoL after treatment initiation is given in Table 6.

**Table 6:** Degree of improvement of parameters assessing the wellbeing and the quality of life associated with treatment with levothyroxine (group 2) or levothyroxine and progestogens or GH (group 3). To assess changes associated with treatment, a 3- (-1, 0, 1) or 5-point rating scale (-2, -1, 0, 1, 2) was used (-2 = lot worse, -1 = little worse, 0 = no change, 1 = little better, 2 = much better). Comparison between groups was performed by using Wilcoxon-rank-sum test or t-test.

	Group 2	Group 3	Comparison between groups p-value^1^
**Change of food intake (-2–2)**	mean: 0.23	mean: 0.73	*Wilcoxon rank sum test*:*0*.*170*
	n^2^ = 22	n = 11	
**Change of playful behaviour (-2–2)**	mean: 0.57	mean: 0.8	*Wilcoxon rank sum test*:*0*.*505*
	n = 21	n = 10	
**Change of quality of life (-2–2)**	mean: 1	mean: 1.31	*Wilcoxon rank sum test*:*0*.*433*
	n = 20	n = 13	
**Change of walking duration/24 hrs (-1–1)**	mean: 0	mean: 0.44	*Wilcoxon rank sum test*:*0*.*060*
	n = 17	n = 9	

^1^p = level of significance

^2^n = number.

In 3/13 treated dogs in group 3 treatment was discontinued as body growth was considered to be sufficient. Two dogs were treated with GH and one with progestogens.

## Discussion

The questionnaire was answered by owners of both dwarf and normal-sized GSD. As both untreated and treated dogs with dwarfism (presumably PD) were included and different treatment strategies were pursued, dogs were assigned to different groups to assess the impact of treatment. A control population of unaffected GSDs was included to compare diseases common in this breed. In this study, data of 47 GSD with dwarfism (presumably PD) were analyzed by assessing signalment, shoulder height, body weight, concurrent diseases, wellbeing, QoL, survival times, performed diagnostic procedures, applied treatment, treatment success, and adverse effects. This is the first study that includes such a large number of dwarfs.

The results showed in general, dwarfs are small and have lower body weights, often suffer from dermatological problems and are less fit compared to GSD not affected by dwarfism. Four dwarfs were diagnosed with CKD (two untreated and two treated with levothyroxine) in comparison to zero recorded cases in the control population and dwarfs of group 3. Treatment with the combination of levothyroxine and progestogens or GH is an effective treatment to promote growth and weight gain and may prevent the development of CKD as no CKD cases were recorded in group 3. Any treatment strategy (levothyroxine vs. levothyroxine and progestogens or GH) seems to prolong survival and there was no difference between those treatments. Adverse effects are uncommon and occur equally in both treatment options.

Since dogs in group 3 were significantly taller and heavier compared to all other dwarfs, the effect of GH or progestogens on body growth is proven. However, normal body size was only reached in one of those dogs (female dog treated with GH). In group 0 the smallest dog was a male GSD with 50cm shoulder height at the age of 5 months. Seven dwarfs were taller ≥50cm of which all were older than 1 year of data collection. One dog in group 1 was a female GSD with a shoulder height of 50cm (in this dog GH had been measured). In group 2, one dog was a male GSD with 52cm shoulder height and in group 3, five dogs (38.5%) were ≥50cm: the tallest dogs had 55cm (female) shoulder height (within breed specific range), four dogs were male with 50cm (two dogs), and one with 53cm and 54cm each. All of them were older than 1 year at the time of data collection. The federation cynologique internationale `breed standard`declares a minimal shoulder height of 55cm in female GSD and 60cm in male GSD [23]. It is possible that the combination treatment was not well titrated in those dogs to reach maximal response–but the dog´s age at which the treatment has begun could also play a role. The prognosis for children with GH deficiency is usually good with reaching improved or even normal heights in treated children [24]. In dogs, breed size has a significant impact on when the growth plates close as well as the anatomic location of the epiphysis. The proximal tibia and proximal humerus are the last long bone epiphyses to close. The proximal tibia in large breed dogs close at 10–18 months and are less responsive if treatment is started thereafter [21, 25]. Treatment with levothyroxine alone does not seem to induce significant growth, although thyroid hormones also play a role in the process of growing and dogs with congenital hypothyroidism may present clinically as dwarfs [26]. Most dogs were adult at the time of enrollment and had probably achieved their maximum height. Only dogs of group 1 had more juvenile dogs in its population, which had a potential for growth. In GSD with PD most dogs are described as being proportionally smaller [5, 17, 27], whereas the term “dwarf” in humans typically refers to an unproportional stature with relatively shortend limb length. More owners of untreated dogs (group 1) described their dog as having an unproportionate stature, whereas treated dwarfs were considered proportionate by their owners. It is possible that treatment had influenced a more proportional development.

In dwarfs, both sexes were accounted equally, indicating that there is no sex predisposition of this disease, which is in accordance with the literature [27].

Overall, many GSD in this study suffered from concurrent disease. In controls, orthopedic and dermatologic problems as well as malignant tumors were common (> 10% of dogs). Five control dogs died or were euthanized due to neoplasia and four of them were older than 10 years. Endocrine diseases were rare and none of the controls suffered from CKD. In the dwarf population, orthopedic and dermatologic diseases were also common, as well as CKD. Most of the dwarfs were dead before the age at which the malignancies developed in the control population. In earlier studies, the most common symptoms of GSD with PD described are growth retardation, abnormal hair coat (lack of primary hair and predominant secondary hairs), bilaterally symmetrical alopecia, hyperpigmentation, and pyoderma. Dogs in group 2 had the highest prevalence of dermatologic problems. It is unlikely that this reflects an adverse effect of the treatment itself as thyroxine supplementation is usually well tolerated [28]. Only in rare cases, hypersensitivity reactions affecting the skin have been demonstrated where inactive ingredients of the levothyroxine tablet were the suspected cause [29], however as there was no question to evaluate either the onset of signs it is not possible to draw any conclusion to the underlying aetiology of the skin problems and GSD`s are a breed predisposed for atopy and pyoderma [30].

In this study 11 dwarfs had orthopedic problems, mainly incomplete atlanto-axial ossification. In an earlier study, atlanto-axial malformation in GSD with PD was reported in 3 Czechoslovakian wolfdogs and 1 GSD with dwarfism which were presented with neurological signs. The Czechoslovakian wolfdog dwarfs had tetraparesis, proprioceptive ataxia and a hypermetric gait. The GSD dwarf had mild paraparesis and mild proprioceptive ataxia of the pelvic limbs, but no hypermetric gait. Diagnostics showed that they had abnormalities of the atlanto-axial joint with instability and dynamic compression of the spinal cord by the dens axis. The authors suggested an association between the LHX3 mutation in dogs with combined pituitary hormone deficiency and atlanto-axial malformations [31]. One dwarf in this study (group 2) had a hypermetric gait, but as no further tests had been performed, it is unclear if an incomplete atlanto-axial ossification was the underlying cause. One patient in group 2 and 1 patient in group 3 had seizures, also with no further tests performed. GSD are frequently affected by idiopathic epilepsy [32].

As reported in the literature, PD may be associated with persistent ductus arteriosus (PDA) [5, 27] and four dwarfs in this study population had been diagnosed with PDA. One other patient in group 2 had a heart murmur of unknown cause. PDA was not detected in unaffected controls.

It is notable that four dwarfs (2 in group 1 and 2 in group 2) in this study were diagnosed with CKD which was, in comparison, reported in 7/8 dogs in one study [5]. A reduced glomerular filtration rate is considered common in dwarfs [5, 12–14]. None of the treated dwarfs of group 3 nor controls were affected by CKD. Therefore, it is possible, that treatment with GH or progestogens is protective. The undertaken treatment strategy of dogs in group 3 can therefore be considered successful. However, the overall low case number of dogs with PD precludes a definitive conclusion.

Considering wellbeing and QoL, the study revealed some surprising findings. As expected, unaffected dogs recorded the highest scores with regard to food intake, playful behaviour and quality of life. The aim of the questionnaire to assess fitness based on the total duration of walks which was also best in this group, unfortunately did not assess how far dogs could walk before they get tired. Untreated dwarfs scored surprisingly well in all categories and treated dogs did not score better, independent of the treatment strategy. According to the owners, treatment does not improve wellbeing and QoL.

In one human study where the QoL of children with disabilities was compared to the QoL of healthy children, children with physical impairments and chronic pain had a lower QoL compared to healthy children and children without physical impairment but other disabilities scored equal to healthy controls [33]. As most dogs with PD have a normal gait and do not exhibit obvious pain, they may appear „normal”to the owner and wellbeing and QoL might be assessed as being comparable to dogs without PD.

Assessment of QoL in dogs with chronic diseases such as atopic dermatitis, Cushing’s syndrome, cancer and idiopathic epilepsy reflects the owner´s perspective and their normative idea of what constitutes a good life. In the afore mentioned human study the child`s perspective of their QoL was not necessarily correlated to that of their parents. However, assessment of wellbeing and QoL in addition to objective parameters (such as clinical and laboratory parameters) are important measures in humans and pets to assess treatment response for many chronic diseases [34–39].

Although treatment failed to change the well-being and QoL in dwarfs, combination treatment with thyroxine and GH or progestogens induced growth. This was the only significant difference between both treatment strategies. Although not statistically significant, more owners of group 3 dogs rated the improvement of the hair and skin condition, change of food intake, and playful behavior higher than owners of group 2 dogs. The improvement of fitness assessed by the walking ability in 24 hours was borderline significant in dogs of group 3. Although not statistically significant the parameters of improved growth (although only one dog reached a normal body size) and lack of reported CKD recorded in group 3 suggest an overall better outcome with the combination treatment with thyroxine and GH or gestagens.

Adverse effects were rare in this study with 14–15% in both treatment groups. Three dogs in group 2 and 2 dogs in group 3 were reported. In dogs treated with levothyroxine persistent estrus and pyoderma were reported. In dogs treated with progestins, pyometra with mastitis and mammary tumor with pyoderma were reported. As stated previously, GSD are predisposed to pyoderma [30]. Hypersensitivity reactions (e.g. angioedema), carbohydrate intolerance, pruritic pyoderma, development of mammary tumors and also development of acromegaly are known adverse effects of progestogens treatment [21, 40, 41]. As cystic endometrial hyperplasia with mucometra is also a known adverse effect ovariohysterectomy is recommended prior to progestin therapy [12]. Diabetes mellitus, another reported side effect of GH and gestagens, was not recorded in this study.

Information regarding the long-term prognosis of treated and untreated dwarfs is sparse. Untreated dwarfs typically die or are euthanized at an early age (less than 5 years of age) [21]. They are usually bald, dull and thin by the age of 3–5 years. The underlying mechanisms may include a progressive loss of pituitary function, progressive renal failure and the expansion of pituitary cysts. Euthanasia is usually requested by the owner at that point [25, 27]. The results of this study are in accordance with this. Out of 5 deceased untreated dogs, 4 died or were euthanized in their first year. Only one reached the age of 7 years. Treatment does apparently prolong survival time. In group 2 the four dead dogs died or were euthanized between 7–13 years. The other 18 dwarfs were recorded as still alive and between 6 months and 13 years of age. In group 3, 5/13 dogs died or were euthanized at the age ranging between 9 months to 7 years. The 8 dogs that were still alive were between 5 and11 years of age. The authors conclude that the most important treatment strategy to ensure longevity of dwarfs with suspected PD is supplementation with levothyroxine.

There are some limitations that need to be considered. Results of the study were based exclusively on the records and perception of owners. Medical data (e.g. diagnosis, treatment procedure, side effects and cause of death) could therefore be imprecise in some patients. Assessment of the overall wellbeing and QoL of dogs are subjective data and under- or overestimations are possible. Especially owner´s, who elected treatment for their dog, could assess treatment response more optimistic (placebo effect).

It cannot be excluded that some dogs in this study suffered from another cause of growth retardation as genetic testing, GH stimulation test, IGF-1 measurement and other diagnostic findings were not performed or available in all dogs. Especially in group 1, in more than 50% of dogs the diagnosis was only tentative.

## Conclusion

In conclusion, the results of this study show the importance of treatment on the overall survival time of dogs with PD. Combination treatment with thyroxine and GH or progestogens will induce growth and possibly prevent the development of CKD. Wellbeing and QoL of treated GSD with PD did not reach scores of unaffected controls but had improved during treatment.

According to this study, all dogs with dwarfism should be treated at the very minimum with levothyroxine to increase the overall survival time.

## Supporting information

S1 File Questionnaire in English. (DOCX)

S2 File Raw data table of the 141 questionnaires answered by participating dog owners. (XLSX)

S1 Table Specification of underlying diseases in control GSD (group 0), untreated dwarfs (group 1), dwarfs treated with levothyroxine (group 2), and dwarfs treated with levothyroxine and progestogens or GH (group 3). (DOCX)

S2 Table Cause of death or euthanasia in control GSD (group 0), untreated dwarfs (group 1), dwarfs treated with levothyroxine (group 2), and dwarfs treated with levothyroxine and progestogens or GH (group 3). (DOCX)
