# Supplementary material for: Wellbeing, quality of life, presence of concurrent diseases, and survival times in untreated and treated German Shepherd dogs with dwarfism
Source: PLoS One. 2021 Aug 9;16(8):e0255678. doi: 10.1371/journal.pone.0255678 (PMC8351940; doi:10.1371/journal.pone.0255678)
Supplement: S1 File — (DOCX) [file pone.0255678.s001.docx]

**Dear dog owner,**

We would like to ask for your help in finding out more information on a specific condition, hereditary dwarfism, that affects German Shepherd dogs. If you can help us then your participation through this questionnaire would really help the breed and aid in allowing us to understand more about this unusual condition and how we can best treat affected dogs. Therefore the purpose of this survey is to gain more scientific data concerning hereditary dwarfism in German Shepherd dogs. This disease is caused by a genetic defect. This defect has already been investigated and we would like to now obtain more data concerning the disease pattern. Therefore, we are looking for German Shepherd dogs that are affected by dwarfism and also unaffected German Shepherd dogs with a normal body size that will act as our control group.

If you are willing then this questionnaire can also be completed if your pet has sadly already passed away.

We really appreciate your help!

All data will be confidential and will be evaluated anonymously. Completion of this questionnaire will be counted as a consent to participate in this study.

Thank you very much for taking your time to fill in this survey!

Stefanie Kitzmann, DVM

Clinic of Small Animal Medicine

Ludwig Maximilian University
Veterinaerstrasse 13
80539 Munich

Germany

E-mail: stefanie.kitzmann@campus.lmu.de

**General part**

**Personal data** (Please do only fill in if you consent, that we may contact you. Evaluation will be anonymous and all personal data will be deleted. It is also possible to participate anonymously by not filling in this section)

| Family name, first name |  |
| --- | --- |
| Address (Please provide your country of residence) |  |
| Telephone number (fixed line). Please can you indicate a good time we could call you on most days |  |
| Telephone number (mobile). Please can you indicate a good time we could call you on most days |  |
| E-mail |  |

**Data of your dog**

| Pet name |  |
| --- | --- |
| Species | Dog |
| Breed | German Shepherd dog |
| Age (in years) |  |
| Date of birth |  |
| Gender | □ Female□ Male |
| Spay or neuter | □ Yes, at the age of:__________________________________□ No |
| Shoulder heights  *(please measure from the point on the back that is at the level of the shoulder blade straight to the ground, while the dog is standing upright)* | ____________cm (measured)  ____________cm (estimated, if measuring is not possible) |
| Body weight | ______ kg (weighed on scales)  ______ kg (estimated, if weighing is not possible) |

**Health status of your dog**

| Known chronic diseases  (more than one answer is  possible) | □ Joint disease: e.g. arthropathy like dysplasia of the elbow,dysplasia of the hips or others□ Chronic skin problems: e.g. pyoderma (bacterial infection of theskin) or others□ Eye abnormalities: e.g. keratitis (typical darkening of the cornea) orothers □ Pancreatic insufficiency □ Chronic gastrointestinal disease□ Neurological disease: e.g. degenerative myelopathy or others□ Chronic kidney disease (if so, when did it occur? ________________)□ Malignant disease (cancer)□ Heart disease□ Other: ____________________________________________□ None |
| --- | --- |
| If your dog already has a known disease, was it treated or is it geting treated now? | □ No□ Yes, the following disease:____________________________________________ ____________________________________________ |
| How would you describe the average food intake of your dog?  (only one answer is possible) | □ Very good (he is always hungry and searching for food)□ Good (he always eats his food, thereafter satiety occurs)□ Normal (sometimes he doesn’t eat the complete food portion)□ Reduced (the daily food intake is reduced, though treats are accepted)□ Poor (the daily food intake is severely reduced, even treats arerefused)□ No food intake |
| How would you describe the average need of your dog to exercise?  (only one answer is possible) | □ Strong interest□ Good interest□ Moderate interest□ Very little interest□ Poor interest□ No interest |
| How would you describe the average playing behaviour of your dog?  (only one answer is possible) | □ Strong interest (brings or fetches the toy several times a day, you canalways encourage him easily)□ Good interest (brings or fetches the toy approximately once a day, ishappy to run)□ Moderate interest (likes to play, but loses interest after some time)□ Very little interest (needs to be encouraged, loses interest quickly)□ Poor interest (can only rarely be encouraged)□ No interest (cannot be encouraged) |
| How would you describe the average social behaviour of your dog towards other dogs?  (only one answer is possible) | □ Very good (he is happy about every other dog, immediatelyenthusiastic)□ Good (he gets along with almost every other dog)□ Satisfying (he has a group of friends, other dogs get ignored)□ Indecisive (he is happy about seeing his friends, but defends histerritory)□ Poor (he only gets along with a few dogs, he tries to stay out of otherdogs‘ way)□ Not interested in other dogs (he is a loner) or aggressive towards otherdogs |
| How do you describe the average sleeping behaviour of your dog?  (only one answer is possible) | □ Normal□ Occasionally anxious during the night (e.g. wandering around,vocalisation)□ Frequently anxious during the night (e.g. wandering around,vocalisation)□ Never able to rest, always wandering |
| How would you describe the general behaviour of your dog?  (only one answer is possible) | □ Very alert and responsive (he doesn’t miss anything going on in his  environment)  □ Alert and responsive (but he is able to focus)  □ Calm (but interested if something new is going on in his environment)  □ Very calm (takes only a little part in his environment)  □ Lethargic (only reacts to strong environmental stimuli) □ Very lethargic (no reaction at all environmental stimuli) |
| How often (average) during 24 hrs do you take your dog for a walk?  (only one answer is possible) | □ More than 5 times□ 4 times□ 3 times□ 2 times□ Once□ Not at all (my dog is only going in to our garden/back yard) |
| What is the average total duration of a walk?  (only one answer is possible) | □ Less than 30 min.□ 30-60 min.□ 60-90 min. □ More than 90 min. |
| How would you describe the general quality of life of your dog?  (only one answer is possible) | □ Very good□ Good□ Satisfying□ Restricted□ Deficient□ Poor |

| Have you ever heard of the possibility to perform genetic testing for dwarfism in German Shepherd dogs? | □ Yes□ No |
| --- | --- |
| Do you perceive your dog to have had normal growth and to now have a normal body stature?  (only one answer is possible) | □ Yes, he has a normal height and proportion.□ No, he is small, but his stature is proportioned.□ No, he is small and his stature is disproportionate.□ No, he has a normal height, but his stature is disproportionate. |
| Do you currently perceive your dog as being ill?  (please skip this question, if your dog has already sadly passed away) | □ No, my dog seems to be healthy.□ Yes, my dog is ill. Problems are:__________________________________________ |
| Has your dog already been treated for any condition?  If so, with which medication and for how long?  (please skip this question, if your dog has already sadly passed away) | □ No□ Yes, with: ____________________________________________ |

| If your dog has already sadly passed away or had to be euthanized, please indicate: | □ Died at the age of:______________________□ Was euthanized at the age of:______________________ |
| --- | --- |
| Cause of death, if known: | ____________________________________________ |

If you have answered the question concerning the growth of your dog (Do you perceive your dog to have had normal growth and to now have a normal body stature?) with ”No, my dog is small and/or his stature is disproportionate, then please proceed with the following questions in Part B. If you have answered ”Yes” my dog has a normal body size and stature, this survey is now terminated. We would like to thank you for your participation!

**Second part**

| Was your dog tested for dwarfism or was it a suspected diagnosis of your veterinarian?  (more than one answer is possible) | □ Yes, the genetic test was performed.□ Yes, growth hormone was measured.□ Yes, a growth hormone stimulating test was performed.□ Yes, the Insulin-Like-Growth-Factor (Somatomedin C) was measured.□ Yes, the thyroid hormones were measured.□ No, it was a suspected diagnosis of my veterinarian and no further testing was performed. |
| --- | --- |

| If your dog was diagnosed with dwarfism, was it treated? | □ Yes□ No |
| --- | --- |
| If the dwarfism was treated, which medications were used?  (more than one answer is possible) | □ Porcine growth hormone (growth hormone of the pig)□ Gestagens (e.g. medroxyprogesterone acetate)□ Thyroid hormones (e.g. levothyroxin)□ Others:­­­­­­___________________________________________ |
| Do you happen to know the dosage and the application frequency of the medications? | □ ___________________________________________________□ Unknown |
| How often was the dosage adjusted? | □ ___________________________________________________□ Unknown |
| Did you notice an improvement after starting treatment?  If so, what kind of improvement?  (more than one answer is possible)  After which time period did you notice an improvement?  (only one answer is possible) | □ No, there was no improvement at all.□ Yes, the hair coat improved.□ Yes, the skin condition improved.□ Yes, my dog started to grow.□ Yes, my dog became fitter.□ Other: ______________________________________□ After approximately 2 weeks□ After approximately 4 weeks□ After approximately 6 weeks□ After approximately 8 weeks□ After approximately 10 weeks □ After more than 10 weeks |
| Did you observe any side effects?  (more than one answer is possible) | □ No, there were no side effects.□ My dog developed diabetes mellitus.□ My dog developed cancer of the mammary gland.□ My dog developed gigantism with its typicalsymptoms (enlargement of the tongue and the interdental spaces).□ My dog developed problems with the uterus.□ My dog developed a skin irritation/ an itchy skin reaction.□ My dog developed skeletal abnormalities.□ Other:___________________________________________ |
| Was it necessary to discontinue the treatment? | □ Yes- How long after starting the therapy? ____________- Why? ____________________________________________□ No |
| Did you see your veterinarian for regular check-ups? | □ Frequently at intervals of: ________________________________□ Not at all, because ________________________________ |
| Did the food intake of your dog change after starting treatment?  (only one answer is possible) | □ Yes, he ate a lot more than before.□ Yes, he ate a little more than before.□ Yes, he ate a little less than before.□ Yes, he ate a lot less than before.□ No, there was no change at all.□ Cannot be assessed. |
| Did the need of your dog to exercise change after starting treatment?  (only one answer is possible) | □ Yes, he exercised a lot more than before.□ Yes, he exercised a little more than before.□ Yes, he exercised a little less than before.□ Yes, he exercised a lot less than before.□ No, there was no change at all.□ Cannot be assessed. |
| Did the average playing behaviour of your dog change after starting treatment?  (only one answer ispossible) | □ Yes, he played a lot more than before.□ Yes, he played a little more than before.□ Yes, he played a little less than before.□ Yes, he played a lot less than before.□ No, there was no change at all.□ Cannot be assessed. |
| Did the social behaviour of your dog towards other dogs change after starting treatment?  (only one answer is possible) | □ Yes, he was a lot more social than before.□ Yes, he was a little more social than before.□ Yes, he was a little less social than before.□ Yes, he was a lot less social than before.□ No, there was no change at all.□ Cannot be assessed. |
| Did the sleeping behaviour of your dog change after starting the treatment?  (only one answer ispossible) | □ Yes, he slept a lot more comfortably than before.□ Yes, he slept a little more comfortably than before.□ Yes, he slept a little more (a little restless) than before.□ Yes, he was more restless than before, he slept less.□ No, there was no change at all.□ Cannot be assessed. |
| Did the general behaviour of your dog change after starting the treatment?  (only one answer is possible) | □ Yes, he became a lot more alert and responsive.  □ Yes, he became a little more alert and responsive.  □ Yes, he became a little calmer.  □ Yes, he became a lot calmer. □ No, there was no change at all. □ Cannot be assessed. |
| Did the frequency of the walks with your dog change after starting the treatment?  (only one answer is possible) | □ Yes, we could go for more walks each day.□ Yes, we could go for less walks each day.□ No, there was no change at all.□ Cannot be assessed. |
| Did the average duration of the walks change after starting treatment?  (only one answer is possible) | □ Yes, we could walk our dog a lot longer (approximately we now walk a total of 1 hour).□ Yes, we could walk our dog a little longer (approximately we now walk a total of 30 min).□ Yes, we had to shorten our walks a little (by approximately 15 min.).□ Yes, we had to shorten our walks a lot (by approximately 30 min.).□ No, there was no change at all.□ Cannot be assessed. |
| Did the quality of life of your dog change after starting the treatment?  (only one answer is possible) | □ Yes, it improved a lot.□ Yes, it improved a little.□ Yes, it became a little worse.□ Yes, it became a lot worse.□ No, there was no change at all.□ Cannot be assessed. |

| For how long has your dog survived after the diagnosis was established?  (only one answer possible) | □ Less than 1 year□ 1-2 years□ 2-3 years□ 3-4 years□ 4-5 years□ More than 5 years, even _______ years |
| --- | --- |

| If your dog suffers/suffered from dwarfism, may we contact your veterinarian for further information (e.g. laboratory results, etc.)?  (Attention!!! If your answer is *YES*, then we will need your contact details and the name of your dog) | □ No□ Yes, name, telephone number and address of my veterinarian are: _____________________________________________________  _____________________________________________________  _____________________________________________________ |
| --- | --- |

In the free text field below additional information that you consider important can be entered.
