# Supplementary material for: Wellbeing, quality of life, presence of concurrent diseases, and survival times in untreated and treated German Shepherd dogs with dwarfism
Source: PLoS One. 2021 Aug 9;16(8):e0255678. doi: 10.1371/journal.pone.0255678 (PMC8351940; doi:10.1371/journal.pone.0255678)
Supplement: S1 Table — (DOCX) [file pone.0255678.s003.docx]

**S1 Table:** Specification of underlying diseases in control GSD (group 0), untreated dwarfs (group 1), dwarfs treated with levothyroxine (group 2), and dwarfs treated with levothyroxine and progestogens or GH (group 3).

| **Specification of diseases** | **Group 0** | **Group 1** | **Group 2** | **Group 3** |
| --- | --- | --- | --- | --- |
| **Dermatologic** | n = 14 | n = 4 | n = 14 | n = 5 |
| Alopecia |  | 3 (one also had pyoderma) | 3 (one also had a history of pyoderma and 1 had current pyoderma) |  |
| Pyoderma | 2 | 1 | 3 | 3 |
| Anal fistulas | 3 |  |  |  |
| Allergy/Atopy | 3 |  |  |  |
| Unknown | 6 |  | 8 | 2 |
| **Orthopedic** | n = 21 | n = 2 | n = 3 | n = 6 |
| Degenerative joint disease | 6 (three also had spondylosis) |  |  | 1 |
| Incomplete ossification of atlas |  |  | 1 | 4 |
| Dysplasia | 3 (one elbow, two hip) |  |  |  |
| Spondylosis | 2 |  |  |  |
| Disc disease | 1 |  |  |  |
| Unknown | 9 | 2 | 2 | 1 |
| **Cardiac** | n = 5 | n = 0 | n = 3 | n = 3 |
| PDA |  |  | 1 | 3 |
| Murmur |  |  | 1 |  |
| Tricuspid dysplasia | 1 |  |  |  |
| Valvular insufficiency | 1 |  |  |  |
| Unknown | 3 |  | 1 |  |
| **Neurologic** | n = 0 | n = 0 | n = 2 | n = 1 |
| Seizures |  |  | 1 | 1 |
| Hypermetric  front legs |  |  | 1 |  |
| **Opthalmologic** | n =1 | n = 0 | n = 2 | n = 1 |
| Keratitis |  |  |  | 1 |
| Unknown | 1 |  | 2 |  |
| **Gastrointestinal** | n = 4 | n = 1 | n = 3 | n = 0 |
| Food-responsive | 3 | 1 |  |  |
| Gastric torsion | 1 |  |  |  |
| Unknown |  |  | 3 |  |
| **Endocrine (besides dwarfism)** | n = 4 | n = 0 | n = 0 | n = 0 |
| Hypothyroidism | 3 |  |  |  |
| Hyperadreno-corticism | 1 |  |  |  |
| **Urogenital (besides CKD)** | n = 4 | n = 0 | n = 0 | n = 1 |
| Cryptorchism | 3 |  |  |  |
| Incontinence (female) | 1 |  |  |  |
| Phimosis |  |  |  | 1 |
| **Respiratory** | n = 2 | n = 0 | n = 1 | n = 0 |
| Deafness |  |  | 1 |  |
| Eosinophilic bronchopneu-mopathy | 1 |  |  |  |
| Laryngeal paralysis | 1 |  |  |  |
| **Neoplasia** | n = 10 | n = 0 | n = 1 | n = 0 |
| Splenic | 2 |  |  |  |
| Lymphoma | 1 |  | 1 |  |
| Osteosarcoma | 1 |  |  |  |
| Testicular | 1 |  |  |  |
| Squamous cell carcinoma | 1 |  |  |  |
| Meningioma | 1 |  |  |  |
| Mammary | 1 |  |  |  |
| Unknown | 2 |  |  |  |
