# Supplementary material for: Wellbeing, quality of life, presence of concurrent diseases, and survival times in untreated and treated German Shepherd dogs with dwarfism
Source: PLoS One. 2021 Aug 9;16(8):e0255678. doi: 10.1371/journal.pone.0255678 (PMC8351940; doi:10.1371/journal.pone.0255678)
Supplement: S2 Table — (DOCX) [file pone.0255678.s004.docx]

**S2 Table:** Cause of death or euthanasia in control GSD (group 0), untreated dwarfs (group 1), dwarfs treated with levothyroxine (group 2), and dwarfs treated with levothyroxine and progestogens or GH (group 3).

| **Death or euthanasia** | **Group 0**  **n =12** | **Group 1**  **n = 5** | **Group 2**  **n = 4** | **Group 3**  **n = 5** |
| --- | --- | --- | --- | --- |
| **Death** |  |  |  |  |
| Incomplete ossification |  |  |  | 2 |
| Dottage | 1 |  | 1 |  |
| Chronic kidney disease |  | 1 | 1 |  |
| Heart failure | 1 |  |  |  |
| Neoplasia | 1 |  |  |  |
| Dwarfism |  | 1 |  |  |
| Unknown |  |  | 1 |  |
| **Euthanasia** |  |  |  |  |
| Neoplasia | 6 |  | 1 |  |
| Dwarfism |  | 3 |  |  |
| Dottage | 2 |  |  |  |
| Gastric torsion | 1 |  |  |  |
| Incomplete ossification |  |  |  | 1 |
| Degenerative joint disease |  |  |  | 1 |
| Unknown |  |  |  | 1 |
